# Implementation of an antibiotic nomogram improves postoperative antibiotic utilization and safety in patients undergoing coronary artery bypass grafting

**DOI:** 10.1186/1754-9493-1-2

**Published:** 2007-11-07

**Authors:** Thomas J Papadimos, Jennifer L Grabarczyk, Daniel F Grum, James P Hofmann, Alan P Marco, Sadik A Khuder

**Affiliations:** 1 Department of Anesthesiology, University of Toledo, College of Medicine, Toledo, USA; 2 Department of Cardiothoracic Anesthesiology, St. Luke's Hospital, Maumee, USA; 3 Pharmacy Department, St. Luke's Hospital, Maumee, USA; 4 University of Toledo, College of Medicine, Department of Medicine, Toledo, USA

## Abstract

**Background:**

Routine, initial, empiric vancomycin dosing by clinicians in postoperative coronary artery bypass grafting (CABG) patients was identified as a potential patient safety issue in the Cardiovascular Intensive Care Unit (CVICU) because the rate of postoperative acute renal insufficiency (ARI) and average patient Body Mass Index (BMI) > 35 kg/m^2 ^were significantly higher in our institution than those of the Society of Thoracic Surgeons (STS) database. A vancomycin dosing nomogram was derived from the local patient population in the attempt to improve patient safety by convincing clinicians to use an evidence-based approach to vancomycin prescription.

**Methods:**

We analyzed two different treatment strategies that were applied consecutively to an intensive care unit population. CABG patients dosed empirically with vancomycin (group 1, pre-nomogram) were compared with CABG patients dosed using a vancomycin dosing nomogram (group 2, post-nomogram) derived from the hospital population using an Internet program that facilitated creation of a local nomogram. The two groups were analyzed as to age, sex, body mass index, creatinine clearance, and vancomycin dosage using logistic regression and testing for continuous and categorical variables.

**Results:**

Nomogram use decreased the number of patients receiving the customary dose of one gram every 12 hours in those group 2 patients with diminished CrCl as compared with those in group 1 with diminished CrCl (group 2, 2/21 vs. group 1, 14/21, p < .0001), as well as in those with a normal creatinine clearance, (group 2, 2/15 vs. group 1, 26/34, p < .0001). Therefore, nomogram use affected the customary dose of one g vancomycin every 12 hours between the two groups overall (group 1, 40/55 vs. group 2, 4/36, p < .001), whereby 32/36 (88.9%) of group 2 patients had their dosing altered when compared to what would have been formerly prescribed, p < .0001. Furthermore, nomogram use resulted in fewer doses of antibiotics per year resulting in a cost savings to the hospital with no increase in the rates of infection.

**Conclusion:**

Implementation of the nomogram resulted in a more appropriate antibiotic utilization, regardless of creatinine clearance, that decreased costs without increasing infection rates.

## Introduction

Vancomycin is used in antibiotic prophylaxis in penicillin-allergic patients and postoperatively to treat life-threatening, multi-drug resistant gram positive infections. Complications with vancomycin use include renal dysfunction [1], inappropriate dosing in obese patients [2] and ototoxicity [3]. In view of these potential complications, the initial, routine, empiric dosing of one gram (g) of vancomycin intravenously (IV) every 12 hours in postoperative CABG patients was identified as a patient safety issue at our institution for two reasons. A larger than expected percentage of these patients had (1) increased rates of postoperative ARI (increase in serum creatinine to greater than 2.0 mg/dl, or ≥ 50% increase in creatinine over baseline preoperative value) [4] and (2) a BMI > 35 kg/m^2^. Both exceeded the norms of the STS database [5]. We determined that there was a need for a standardized approach to vancomycin dosing by physicians in order to avoid complications.

The 2006 Disease-Specific Care National Patient Safety Goals of the Joint Commission on Accreditation of Health Care Organizations included the objective (goal #3) of improving the safety of medication use [6]. In support of this goal we report an intervention by a critical care team representing pharmacy and anesthesiology that implemented a mandatory, evidence-based program for vancomycin administration in which a locally derived nomogram was utilized for dosing, as opposed to empiric administration by clinicians. The nomogram is unique because it was derived from the hospital's database with the intent to be specific and continuously adjustable using ongoing data collection. Although generic vancomycin dosing nomograms are available [7-9], we anticipated that (1) the development of a locally derived nomogram would help clinician acceptance of a unified approach to antibiotic prescribing by eliminating the "not invented here" syndrome where external experience is erroneously discounted, and (2) mandatory implementation of this nomogram would correct clinicians' empiric prescribing habits by providing an evidence-based method for dosing vancomycin that would result in more appropriate antibiotic prescription and utilization, thus enhancing patient safety.

## Methods

A locally developed vancomycin nomogram was implemented as part of a performance improvement project in the CVICU of an academically affiliated hospital. Institutional Review Board approval was obtained. The CVICU differed from the hospital's general medical-surgical intensive care unit in that it had (1) a multidisciplinary care team comprised of anesthesiology, surgery, nursing, pharmacy, and respiratory therapy that rounded daily on the patients, and (2) it had a dedicated data collection system. Starting 1 December 2003 a consecutive sample of patients who had CABG surgery and received empiric vancomycin (group 1, n = 55) was compared with a consecutive sample of CABG patients who received vancomycin dosing by nomogram (group 2, n = 36). The nomogram was derived using data from 75 consecutive prior CABG patients who had received an initial vancomycin-loading dose of 15–20 mg/kg, and five subsequent maintenance doses of 10–15 mg/kg. The trough and peak levels were analyzed to determine each patient's pharmacokinetic profile, as required by the Internet support program, GlobalRPh, which was used to create the hospital nomogram [10]. Thus, a nomogram was created by the pharmacy specifically for patients at our institution that differed from previously published generic nomograms [7-9]. ARI was defined in this study as a creatinine clearance (CrCl) < 60 milliliters/minute, according to the American Kidney Foundation guidelines [11]. The serum creatinine level was not used as a marker of ARI because the STS had been considering a revision of the definition [4]. All vancomycin doses were given IV.

Statistical analysis included use of t-testing with the assumption of equal variances for continuous variables, and Fisher exact test for categorical variables. A logistic regression model was used to test for differences in CrCl between groups 1 and 2 and for adjusting for the confounding effect of age and sex. All analyses were done using SAS 9.1 (SAS Institute, Cary NC). P-values < 0.05 were considered statistically significant.

## Results

The pre-nomogram (group 1) and post-nomogram (group 2) groups were similar for age (65.7 vs. 64.2), male sex (54.6% vs. 61.1%), and BMI (29.2 kg/m^2 ^vs. 28.8 kg/m^2^) (Table 1). However, when age and sex were compared with creatinine clearance (regardless of group) there was a difference in that those with a decreased creatinine clearance were older (70.1 years vs. 60.8 years) and female (52.4% vs. 34.7%). A logistic regression of these variables was determined and returned a p value of 0.017 (Table 2).

**Table 1:** Equivalence of parameters between groups

	Pre-nomogram	Post-nomogram	p-value
Age (mean ± sd)	65.7 ± 12.3	64.2 ± 11.7	0.554
Gender % female	45.5	38.9	0.536
BMI (mean ± sd)	29.2 ± 5.4	28.8 ± 6.6	0.774

sd = standard deviation; P < .05 was considered statistically significant

**Table 2:** Logistic regression variables

	Estimate	p-value	OR	95% CI
Group	0.60	0.017	3.34	1.24–9.03
Gender	-0.48	0.053	0.38	0.15–1.01
Age	-0.08	0.0001	0.92	0.88–0.96

OR = odds ratio; Cl = confidence limits; P < .05 was considered statistically significant

In group 1, 40/55 (72.7%) patients received an initial empiric dosing of one g of IV vancomycin every 12 hours while the other 15 patients received doses ranging from 0.5 g IV every 12 hours to 1.25 g every 48 hours. Physicians dosed all 55 patients in a self-determined manner. Using the nomogram, only 4/36 (11.1%) of group 2 patients received the customary vancomycin dose of one g IV every 12 hours. Therefore, 32/36 (88.9%) of group 2 patients had their dosing altered when compared to what would have been formerly prescribed, p < .0001.

The effect of the nomogram was also examined on subsets of patients according to their CrCl. Group 2 had a higher percentage of patients with a decreased CrCl, 21/36 (58.3%) vs. 21/55 (38.2%), p < .0001. In group 1 14/21 (66.7%) of patients with a decreased CrCl received an initial regimen of one g vancomycin IV every 12 hours compared to 2/21 (9.5%) in group 2 patients with a decreased CrCl, p < .0001. Additionally, even those with a normal CrCl exhibited decreased dosing of one g vancomycin IV every 12 hours post-nomogram, 26/34 (76.5%) vs. 2/15 (13.3%), p < .0001.

**Figure 1:**
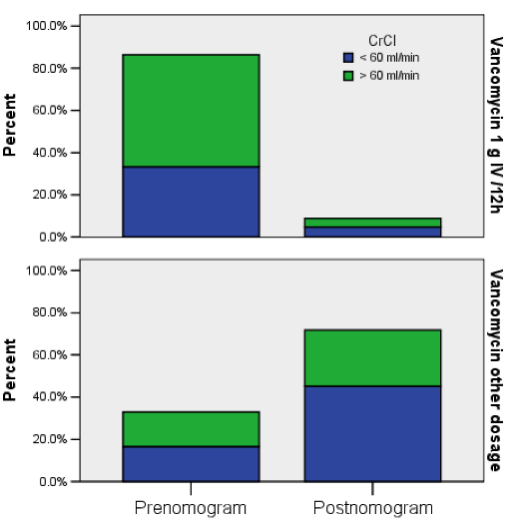
Creatinine clearance (CrCl) vs. vancomycin dosage in group 1 (pre-nomogram) and group 2 (post-nomogram) by percentage of patients receiving one gram intravenously every 12 hours, or a dosage other than one gram intravenously every 12 hours.

Figure 1 demonstrates that 40/44 (90.9%) patients who received one g vancomycin IV every 12 hours were in the pre-nomogram group, whereas prescription of this vancomycin dosage decreased in post-nomogram group, 15/47 (31.9%), p < .0001. There was no significant difference in the number of antibiotic trough levels performed, or in the number of elevated troughs in the two groups, regardless of CrCl. All clinicians prescribing antibiotics in the CVICU complied with use of the nomogram without objection. Infection rates were not affected by the nomogram when comparing years 2001–2003, collectively, with year 2005 (Table 3). Based upon the favorable results shown by a review of the data, use of the nomogram was then instituted as part of routine practice in the CABG population. This practice was then extended hospital-wide and resulted in a reduction of the annual number of doses by 1200.

**Table 3:** Infection rates by site

	2001–2003 (n = 504)	2005 (n = 219)	p-value
Leg	14(2.78%)	2(0.91%)	0.117
Sternum	1(0.20%)	0(0.0%)	0.510
Urinary tract	9(1.79%)	4(1.82%)	0.969
Pneumonia	15(2.98%)	4(1.90%)	0.375
Septicemia	7(1.39%)	3(1.20%)	0.984

P < .05 was considered statistically significant

## Discussion

Cardiac surgery patients have perioperative risk factors such as renal failure and obesity that may influence vancomycin dosing and clearance [1,2]. The percentage of CABG patients with increased levels of serum creatinine and obesity was a concern because the patients' increased postoperative serum creatinines, or ARI, exceeded that of the STS database, 6.75% vs. 3.47% (p < .001), as did their BMI > 35 kg/m^2^, 20.4% vs. 13.4% (p < .001) [5]. Ototoxicity is a known complication of vancomycin therapy and was not addressed by this study. However, since overall dosage decreased for the hospital population, it could be presumed that this risk decreased.

The routine prescription of one g of vancomycin IV every 12 hours, without initially taking into consideration the patient's CrCl, was a prescribing habit that was corrected through the creation of a locally derived nomogram. The nomogram changed the dose that would have been formerly prescribed in 88.9% of group 2 patients. This was evident in those with and without increased CrCl. In view of the fact that those with decreased CrCl were older, and that group 2 had a significantly increased percentage of patients with a decreased CrCl, it may be inferred that they benefited from use of the nomogram.

There are generic vancomycin dosing nomograms that are available and well-validated [7-9]. However, our nomogram's creation and implementation tailored time intervals and doses to our population's CrCl that differed from the generic versions. This convinced physicians to eliminate their empiric vancomycin dosing, thereby allowing more appropriate antibiotic prescription and utilization. Ready physician acceptance of this format occurred because of the potential for increased safety, and because pharmacy calculated the CrCl and antibiotic dose through the locally derived nomogram which facilitated the clinicians' practice. The nomogram's application hospital-wide resulted in excess of $80,000 in annual savings (1200 vancomycin doses per annum at $70 per dose).

There is a growing body of evidence that an individual's genetic background (pharmacogenetics) can influence responses to medications (the metabolism of codeine is a classic example) [12]. Additionally, a local population also might differ enough from the national norm, by ethnicity for example, so that a locally derived nomogram would be appropriate [13]. The fact that in Ohio 60.2% of the population is overweight or obese lends further support to local nomogram use in antibiotic prescription [14]. Therefore a locally derived nomogram tailoring doses and dosing intervals to the the CrCl of a particular population may be of value, not only as an evidenced-based method of antibiotic prescription, but also as a more effective means of treatment. What must be emphasized is that this nomogram intervention caused clinicians to prescribe an antibiotic in a commensurate manner, with pharmacy oversight, resulting in financial savings that did not raise infection rates. The project was not designed to answer the question of whether locally derived nomograms are better than generic nomograms; it was implemented as a performance improvement project. It was a pre-emptive attempt to improve patient safety by convincing clinicians to use an evidence-based approach to vancomycin prescription. Additionally, our institution's ARI did decrease over time by nearly one half to 3.4% (2006) and now approaches the STS database norm. The previously elevated rates of ARI could not be attributed soley to vancomycin, and neither can the current improvement be entirely attributed to use of the nomogram. The hospital staff have made a considerable effort to effect a policy of appropriate hydration in patients, i.e., before and after cardiac catheterization and perioperatively. Therefore, this improvement of ARI must be considered to be multifactorial.

It must be noted that our study design had limitations. We analyzed two different treatment strategies that were applied consecutively to an intensive care unit population. Therefore, it was not randomized, so allocation to treatment groups was not possible. Some specific characteristics other than age, sex, and BMI may have been different between the groups. For example, a history of dysrhythmias, variations in ejection fraction, number and types of vessels bypassed, the types of bypass conduits used (veins vs. arteries), and the duration of cardiopulmonary bypass could affect outcomes. Finally, other factors beside the nomogram may be responsible for the outcome reported, such as the decision of a physician to over-ride the nomogram based on an unusual clinical situation or presentation.

This intervention improved patient care by (1) decreasing the total number of antibiotic doses (risk events) to which the population was exposed, (2) providing a consistent evidence-based method of vancomycin prescription that clinicians incorporated into practice, and (3) promoting a culture of safety. We hope presentation of this successful implementation of an evidence-based method will encourage our colleagues to use this approach as a basis for improved antibiotic utilization and enhanced patient safety in the prescription of medications.

## Competing interests

The author(s) declare that they have no competing interests.

## Authors' contributions

JLG and TJP conceived and designed the project; JGL and TJP acquired the data; TJP, DFG, JPH, and SAK analyzed and interpreted the data; TJP, DFG, JPH, APM, and SAK were involved in drafting the manuscript; all authors (TJP, JLG, DFG, JPH, APM, and SAK) read and approved the final manuscript.

## References

[B1] Gonzalez-Martin G, Acuna V, Perez C, Labarca A, Guevara A, Tagel R (1996). Pharmacokinetics of vancomycin in patients with severely impaired renal function. Int J Clin Pharmacol Ther.

[B2] Penzak SR, Gubbins PO, Rodvold KA, Hickerson S (1998). Therapeutic drug monitoring of vancomycin in a morbidly obese patient. Ther Drug Monit.

[B3] Sorrell TC, Collignon PJ (1985). A prospective study of adverse reactions associated with vancomycin therapy. J Antimicrob Chemother.

[B4] STS National Database Definition Clarification. http://sts.org/doc/4862.

[B5] Papadimos TJ, Habib RH, Zacharias A, Schwann TA, Riordan CJ, Durham SJ, Shah A (2005). Early efficacy of CABG care delivery in a low-procedure volume community hospital: operative and midterm results. BMC Surg.

[B6] No author (2005). The Joint Commission announces the 2006 National Patient Safety Goals and requirements. Jt Comm Perspect.

[B7] Moellering RC, Kogstad DJ, Greenblatt DJ (1981). Vancomycin therapy in patients with impaired renal function: a nomogram for dosage. Ann Intern Med.

[B8] Rybak MJ, Boike SC (1986). Individualized adjustment of vancomycin dosage: comparison with two dosage nomograms. Drug Intell Clin Pharm.

[B9] Lake KS, Peterson CD (1985). A simplified dosing method for initiating vancomycin therapy. Pharmacotherapy.

[B10] Global RPh. http://GlobalRPh.com.

[B11] Bakris GL, Williams M, Dworkin L, Elliot WJ, Epstein M, Toto R, Tuttle K, Douglas J, Hsueh W, Sowers J (2000). Preserving renal function in adults with hypertension and diabetes: a consensus approach. National Kidney Foundation Hypertension and Diabetes Executive Committees Working Group. Am J Kidney Dis.

[B12] Lotsch J, Geisslinger G (2006). Current evidence for a genetic modulation of the response to analgesics. Pain.

[B13] United States Census 2000. http://factfinder.census.gov/servlet/SAFFPeople?.

[B14] Trust for America's Health Reports. http://healthyamericans.org/reports/obesity2005.

